# Legume Shrubs Are More Nitrogen-Homeostatic than Non-legume Shrubs

**DOI:** 10.3389/fpls.2017.01662

**Published:** 2017-09-26

**Authors:** Yanpei Guo, Xian Yang, Christian Schöb, Youxu Jiang, Zhiyao Tang

**Affiliations:** ^1^Department of Ecology, College of Urban and Environmental Sciences and Key Laboratory for Earth Surface Processes, Peking University, Beijing, China; ^2^Department of Evolutionary Biology and Environmental Studies, University of Zurich, Zurich, Switzerland; ^3^School of Biology, Georgia Institute of Technology, Atlanta, GA, United States; ^4^Institute of Forest Ecological Environment and Protection, Chinese Academy of Forestry, Beijing, China

**Keywords:** legume shrubs, non-N_2_-fixing shrubs, nutrient scaling, N:P, stoichiometric homeostasis, symbiotic nitrogen fixation

## Abstract

Legumes are characterized as keeping stable nutrient supply under nutrient-limited conditions. However, few studies examined the legumes' stoichiometric advantages over other plants across various taxa in natural ecosystems. We explored differences in nitrogen (N) and phosphorus (P) stoichiometry of different tissue types (leaf, stem, and root) between N_2_-fixing legume shrubs and non-N_2_-fixing shrubs from 299 broadleaved deciduous shrubland sites in northern China. After excluding effects of taxonomy and environmental variables, these two functional groups differed considerably in nutrient regulation. N concentrations and N:P ratios were higher in legume shrubs than in non-N_2_-fixing shrubs. N concentrations were positively correlated between the plants and soil for non-N_2_-fixing shrubs, but not for legume shrubs, indicating a stronger stoichiometric homeostasis in legume shrubs than in non-N_2_-fixing shrubs. N concentrations were positively correlated among three tissue types for non-N_2_-fixing shrubs, but not between leaves and non-leaf tissues for legume shrubs, demonstrating that N concentrations were more dependent among tissues for non-N_2_-fixing shrubs than for legume shrubs. N and P concentrations were correlated within all tissues for both functional groups, but the regression slopes were flatter for legume shrubs than non-N_2_-fixing shrubs, implying that legume shrubs were more P limited than non-N_2_-fixing shrubs. These results address significant differences in stoichiometry between legume shrubs and non-N_2_-fixing shrubs, and indicate the influence of symbiotic nitrogen fixation (SNF) on plant stoichiometry. Overall, N_2_-fixing legume shrubs are higher and more stoichiometrically homeostatic in N concentrations. However, due to excess uptake of N, legumes may suffer from potential P limitation. With their N advantage, legume shrubs could be good nurse plants in restoration sites with degraded soil, but their P supply should be taken care of during management according to our results.

## Introduction

Symbiotic nitrogen fixation (SNF) is a strategy of plants to acquire nitrogen (N) from the atmosphere, i.e., a trait shared by a large number of species in the Fabaceae family (legumes). SNF broadens potential N resources and generally increases N absorption, resulting in higher N concentration ([N]) and nitrogen to phosphorus (P) ratio (N:P) in N_2_-fixing legumes than non-N_2_-fixing plants (Güsewell et al., 2003). For example, previous studies in the tropics have reported higher leaf [N] in legumes than in other plants (Townsend et al., 2007; Nasto et al., 2014; Bhaskar et al., 2016), which could be related to higher photosynthetic capacity and water use efficiency (McKey, 1994; Adams et al., 2016). SNF also helps legumes to keep stable [N] in N-limited soils (Lambers et al., 2008; Hobbie, 2015). Therefore, N_2_-fixing legumes can be more abundant in N-limited habitats with less competitive exclusion from non-N_2_-fixing plants (Rastetter et al., 2001; Menge et al., 2008). Such an ability of plants to maintain their nutrient composition despite nutrient variation in their resource supplies was regarded as stoichiometric homeostasis (Sterner and Elser, 2002; Elser et al., 2010). In this sense, N_2_-fixing legumes can be more N-homeostatic than non-N_2_-fixing plants.

Because of sufficient N and stronger homeostasis, legumes are also likely to differ from non-N_2_-fixing plants in the nutrient scaling relationship among tissues. [N] and [P] in different tissues are usually dependent and show correlated responses to ecological and evolutionary factors (Kerkhoff et al., 2006; Yang et al., 2014). Soil N availability is the direct cause of plant [N] variation (Elser et al., 2010). However, since soil N availability may not be the driving factor for legumes' [N], there could be less variation and co-variation in [N] among different tissues. For example, Liu et al. (2010) found that root and leaf [N] of a legume *Caragana microphylla* did not co-vary. However, another study found little difference in nutrient scaling relationships between legumes and non-legume plants (Yang et al., 2014). Thus, it is still an open question how SNF affects the nutrient co-variation among tissue types.

Previous studies also found coordinated variation of N and P within tissues (Broadley et al., 2004; Kerkhoff et al., 2006; Townsend et al., 2007; He et al., 2008; Sardans et al., 2016). Taking leaves for example, this correlated change can be described as:

$$\begin{array}{l} {\left[\text{N}\right]}_{\text{leaf}}=a\;{{\left[\text{P}\right]}_{\text{leaf}}}^{b} \end{array}$$

This equation can be ln-transformed (logarithm transformation to the base of the mathematical constant *e*) to:

$$\begin{array}{l} \text{ln}\left({\left[\text{N}\right]}_{\text{leaf}}\right)=\text{ln}\left(a\right)+b\text{ ln}\left({\left[\text{P}\right]}_{\text{leaf}}\right) \end{array}$$

The slope *b* is normally positive, showing the positive relationship between ln-transformed leaf [N] and [P]. Even though there is disagreement over the slope *b* being 3/4 (Kerkhoff and Enquist, 2006; Niklas, 2006) or 2/3 (Reich et al., 2009), the consensus is that [N] increases relatively more slowly than [P] (Wright et al., 2004; Elser et al., 2010). Kerkhoff et al. (2006) demonstrated that N–P relationships can differ between life forms, and Ågren (2008) said that the excess uptake of N or P could shift the slopes of N–P relationships. As a result, we may infer that legumes with sufficient N supply have different N–P relationships from non-N_2_-fixing plants often faced with N limitation. Legumes with very high N:P might suffer from P limitation (Güsewell, 2004), so the slopes of N–P relationships can be lower due to the excess N absorption (Ågren, 2008), i.e., the increasing rate of [P] can be even faster.

In summary, we expected stoichiometry of N_2_-fixing legumes and non-N_2_-fixing plants to differ considerably, and that this would influence plant nutrition responses to the variation in soil and change plant stoichiometric regulation among tissues.

In this study, we compared the stoichiometric patterns of N_2_-fixing legume shrubs and non-N_2_-fixing shrubs in northern China. Compared to trees, shrubs are generally more uniform in size, so the plant size effect and the “dilution” of nutrients in structural tissues of large trees is weaker (Kerkhoff et al., 2006; Elser et al., 2010; Yang et al., 2014). In temperate China, species in the genera *Lespedeza, Caragana*, and *Sophora* are among the most widespread legume shrubs, while species in the genera *Vitex, Corylus*, and *Spiraea* are typical non-N_2_-fixing shrubs. Previous studies have reported the use of legume shrubs in ecological restoration where they act as nurse plants to facilitate the growth of tree seedlings and herbs by increasing soil nitrogen levels (Gómez-Aparicio et al., 2004; Zhao et al., 2007). Our research could provide useful suggestions in legumes' nutritional requirement for this application.

Specifically, we proposed the following hypotheses.

1. N_2_-fixing legume shrubs contain higher [N] and N:P than non-N_2_-fixing shrubs because of their broader sources of N.
2. N_2_-fixing legume shrubs are more stoichiometrically homeostatic in [N], than non-N_2_-fixing shrubs. In other words, [N] are more independent of soil N availability in legume shrubs after controlling for taxonomic and other environmental effects.
3. Different tissues of N_2_-fixing legume shrubs may exhibit weaker co-variation of [N], i.e., [N] among tissues are less correlated for legume shrubs compared to non-N_2_-fixing shrubs when only considering the effect of soil [N].
4. In order to maintain optimal N:P, the increasing rate of [P] relative to [N] in legume shrubs is higher than that in non-N_2_-fixing shrubs, which means the legume shrubs have gentler N–P slopes within tissues than non-N_2_-fixing shrubs.

## Materials and methods

### Study sites and sampling

**Figure 1:**
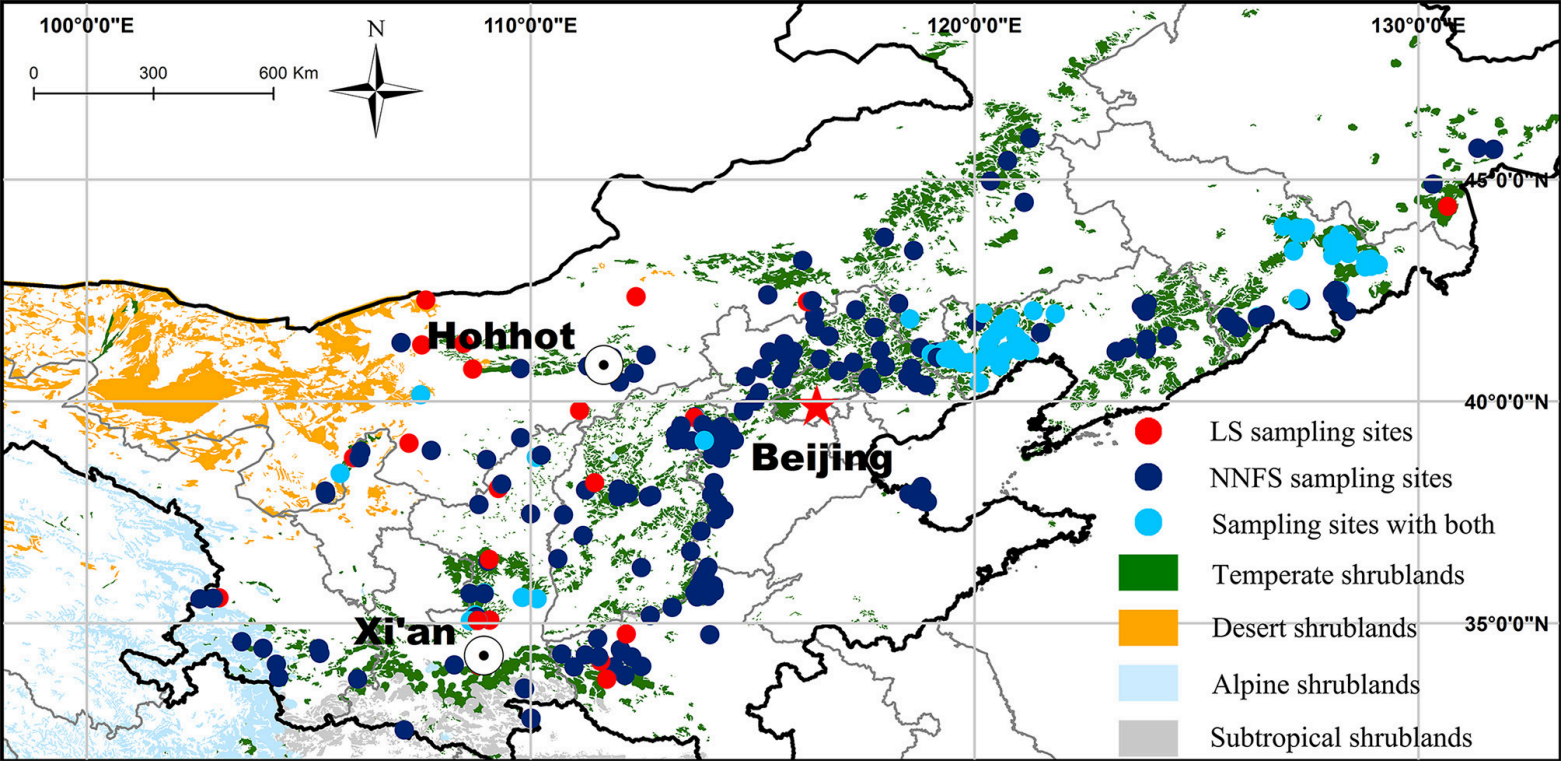
Distribution of the sampling sites with a background of the vegetation in northern China. LS, legume shrubs; NNFS, non-N_2_-fixing shrubs.

Leaf, stem, and root samples of legume shrubs and non-N_2_-fixing shrubs were collected in 299 natural temperate broadleaved deciduous shrubland sites between July and September (mostly July and August) from 2011 to 2013. Among these sites, legume shrubs were sampled in 96 sites each with three 25 m^2^ plots (Figure 1). Totally, we sampled 19 species of six genera in the Fabaceae family with 105 replicates and 113 species of 64 genera in 32 non-N_2_-fixing families with 534 replicates (see Supplementary Data Sheet 2 for details). Species were identified according to the *Flora of China* (Wu et al., 2006). Non-legume N_2_-fixing families such as, Elaegnaceae and Coriariaceae were excluded. We classified plant families by the APG III system (APG III, 2009). At each site, fully expanded sun leaves, stems and roots (mainly coarse roots in the top 30 cm of soil) of at least five individuals of each species were collected and assembled in fabric bags then dried in the sun, transported to the laboratory and oven-dried at 65°C for 72 h.

We also investigated soil nutrient concentrations of the sites. To do this, three one-meter-deep (or deep to the base rock) pits along the diagonal of each plot were excavated to collect soil samples. For each profile, soil at the depth of 0–10, 10–20, 20–30, 30–50, 50–70, 70–100 cm was sampled and soil samples from the same depth in one plot were mixed. We also excavated one profile of 100 cm outside each site and collected soil cores in left, right, and front surfaces at the depth of 0–10, 10–20, 20–30, 30–50, 50–70, 70–100 cm to measure the soil bulk density (BD). Soil samples used to test element concentrations were air-dried, roots removed, and then ground to pass through a 100 μm mesh sieve (see Yang et al., 2014 for more details on the sampling protocol).

Climate variables we used in this analysis include mean growing season temperature (MGT) and growing season precipitation (GP). Growing season was defined from May to October (Yang et al., 2014), and mean monthly temperature and precipitation in growing season were extracted from the means of 1970 to 2000 of the WorldClim spatial climate data (Hijmans et al., 2005; available at http://www.worldclim.org).

### Measurement of nutrient concentrations

[N] and [P] of plant and soil samples were measured at the Measurement Center of the Institute of Botany, Chinese Academy of Sciences. Soil total [N] (STN) and plant tissue (leaf, stem, and root) [N] were determined using an elemental analyzer (2400 II CHNS; Perkin-Elmer, Boston, MA, USA) under 950°C for combustion then reduced to 640°C. Soil total [P] (STP) and plant tissue (leaf, stem and root) [P] were measured using the molydate/ascorbic acid method after H_2_SO_4_-H_2_O_2_ digestion (Jones, 2001). STN and STP from 0 to 30 cm were used as surrogates of the soil nutrient conditions, because STN and STP were highly correlated at all depths, and soil depths in many sites did not reach 100 cm.

### Data analysis

Student's *t*-test was applied to compare the ln-transformed [N], [P], and N:P of each tissue between legume shrubs and non-N_2_-fixing shrubs after comparing their variance by an *F*-test.

In order to remove the influences of species identity, climate (MGT and GP) and other soil factors (BD and the other nutrient concentration), we first calculated the residuals of tissue nutrient compositions ([N]_res_, [P]_res_, and N:P_res_) using general linear models. Taking the leaf as an example, [N]_res_, [P]_res_, and N:P_res_ can be presented as error terms (ε_1_, ε_2_, and ε_3_, respectively) of regression equations.

(1) $$\begin{array}{l} \text{ln}\left(\text{leaf }\left[\text{N}\right]\right)=\alpha_{0}+\alpha_{1}\text{Family}+\alpha_{2}\text{Genus}+\alpha_{3}\text{MGT}+\alpha_{4}\text{GP}\\ +\alpha_{5}\text{BD}+\alpha_{6}\text{ln STP}+\varepsilon_{1} \end{array}$$

(2) $$\begin{array}{l} \text{ln}\left(\text{leaf }\left[\text{P}\right]\right)=\beta_{0}+\beta_{1}\text{Family}+\beta_{2}\text{Genus}+\beta_{3}\text{MGT}+\beta_{4}\text{GP}\\ +\beta_{5}\text{BD}+\beta_{6}\text{ln STN}+\varepsilon_{2} \end{array}$$

(3) $$\begin{array}{l} \text{ln}\left(\text{leaf N}:\text{P}\right)=\gamma_{0}+\gamma_{1}\text{Family}+\gamma_{2}\text{Genus}+\gamma_{3}\text{MGT}+\gamma_{4}\text{GP}\\ +\gamma_{5}\text{BD}+\varepsilon_{3} \end{array}$$

The ε_1_, ε_2_, and ε_3_ stand for the part of nutrient variance that cannot be explained by other effects but corresponding soil nutrient compositions only. Hence, leaf [N]_res_, [P]_res_, and N:P_res_ can be calculated as follows.

(4) $$\begin{array}{l} \text{Leaf}{\left[\text{N}\right]}_{\text{res}}=\text{ln}\left(\text{Leaf}\left[\text{N}\right]\right)-(\alpha_{0}+\alpha_{1}\text{Family}+\alpha_{2}\text{Genus}\\ \;+\alpha_{3}\text{MGT}+\alpha_{4}\text{GP}+\alpha_{5}\text{BD}+\alpha_{6}\text{lnSTP}) \end{array}$$

(5) $$\begin{array}{l} \text{Leaf}{\left[\text{P}\right]}_{\text{res}}=\text{ln}\left(\text{Leaf}\left[\text{P}\right]\right)-(\beta_{0}+\beta_{1}\text{Family}+\beta_{2}\text{Genus}\\ \;+\beta_{3}\text{MGT}+\beta_{4}\text{GP}+\beta_{5}\text{BD}+\beta_{6}\text{lnSTN}) \end{array}$$

(6) $$\begin{array}{l} \text{Leaf N}:{\text{P}}_{\text{res}}=\text{ln}\left(\text{Leaf N}:\text{P}\right)-(\gamma_{0}+\gamma_{1}\text{Family}+\gamma_{2}\text{Genus}\\ \;+\gamma_{3}\text{MGT}+\gamma_{4}\text{GP}+\gamma_{5}\text{BD}) \end{array}$$

Reduced major axis (RMA) estimation was applied to explore the relationship of nutrient concentrations residuals between tissues and soil, among different tissues, and within each tissue. An allometric equation was employed to fit the relationship using the ln-transformed stoichiometric traits.

To explore the homeostasis of plant nutrients, we built an equation between the nutrients in plants and those in soils,

(6) $$\begin{array}{l} \text{ln}\left(\text{Y}\right)=\text{ln}\left(\text{a}\right)+\left(1/\text{H}\right)\text{ ln}\left(\text{X}\right) \end{array}$$

where *X* stands for nutrient compositions in soil and ln(*Y*) stands for [N]_res_, [P]_res_, and N:P_res_ of plant tissues, because the calculation of nutrient residuals has included the ln-transformation of nutrients. The homeostasis coefficient *H*, which equals the reciprocal of the slope, was used to quantify the homeostasis. *H* equivalent to 1 (slope equaling 1) indicates a lack of homeostasis, *H* between 1 and infinity (slope between 0 and 1) indicates incomplete homeostasis, and an infinite *H* (slope equaling 0 or insignificant) means strict homeostasis (Sterner and Elser, 2002; Elser et al., 2010).

We also explored the relationship for nutrients across different tissue types using an exponential scaling approach,

(7) $$\begin{array}{l} \text{ln}\left(Y\right)=\text{ln}\left(a\right)+b\text{ ln}\left(X\right) \end{array}$$

where nutrient residuals of the tissue close to the ground were set as ln(*X*), whereas those of the tissue close to the top were set as ln(*Y*). The equation of nutrient scaling relationships within tissues is still the same, while ln(*X*) and ln(*Y*) represented tissue [P]_res_ and [N]_res_, respectively. The exponential slope (*b*) was used to represent the relative rate of nutrient accumulation. A *b* equivalent to 1 indicates an isometric allometry, whereas *b* different from 1 indicates asymmetric allometry, with *b* > 1 suggesting that *Y* increases more quickly than *X* and *b* < 1 suggesting the opposite (Kerkhoff et al., 2006; Yang et al., 2014). We used likelihood ratio tests when comparing the regression slopes between functional groups.

To test if the patterns are consistent among different taxonomic groups, we refined the non-N_2_-fixing shrubs to a certain taxonomic group by selecting the family (Rosaceae with 150 replicates, to confine all non-N_2_-fixing shrubs to a family) and the genus (*Vitex* with 94 replicates, to confine all non-N_2_-fixing shrubs to a genus) with most replicates. We then repeated the same procedure to examine patterns for species from the family Rosaceae, the genus *Vitex* and the rest (the remaining 290 non-N_2_-fixing shrubs samples from neither Rosaceae nor *Vitex*), respectively, but omitted the “Family” and both “Family” and “Genus” terms when calculating residuals for the Rosaceae and the *Vitex*, respectively.

All analyses were conducted using the basic and smatr (RMA regression and likelihood ratio test) packages of R version 3.2.2 (Warton et al., 2012; R Core Team, 2015).

## Results

### Nutrient concentrations and ratios in legume shrubs and non-N_2_-fixing shrubs

**Figure 2:**
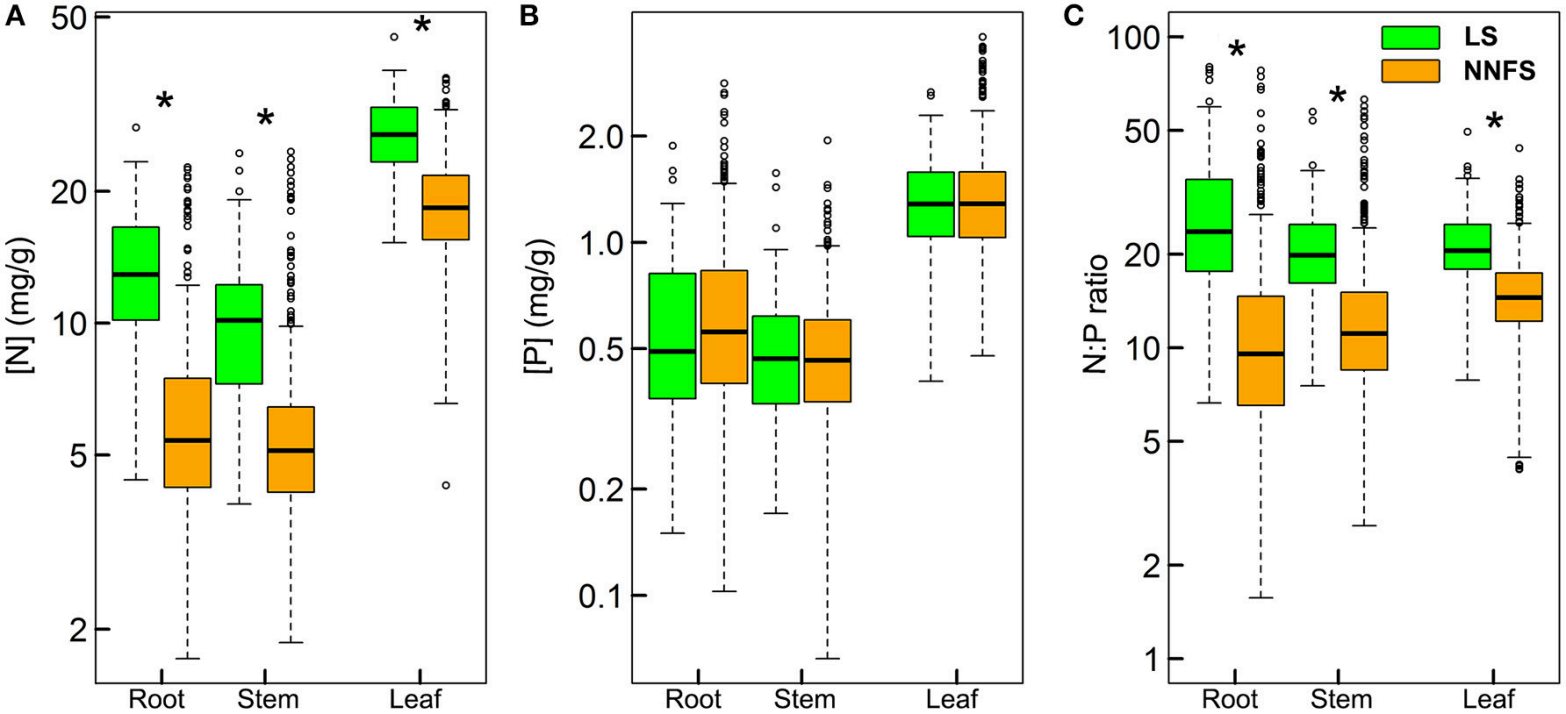
Box-whisker plots showing N **(A)**, P **(B)**, and N:P **(C)** in different tissues of legume shrubs (LS) and non-N_2_-fixing shrubs (NNFS). Asterisks denote significant differences at *p* < 0.05.

Geometric mean [N] (± geometric standard deviation) for leaves, stems and roots were 26.7 ± 1.2, 9.43 ± 1.5, and 12.6 ± 1.5 mg.g^−1^, respectively, in legume shrubs, and 18.3 ± 1.3, 5.3 ± 1.5, and 5.7 ± 1.6 mg.${\text{g}}_{,}^{-1}$ respectively, in non-N_2_-fixing shrubs. Geometric mean [P] for leaves, stems and roots were 1.3 ± 1.4, 0.5 ± 1.5, and 0.5 ± 1.7 mg.g^−1^, respectively, in legume shrubs, and 1.3 ± 1.4, 0.5 ± 1.5, and 0.6 ± 1.7 mg.${\text{g}}_{,}^{-1}$ respectively, in non-N_2_-fixing shrubs. Geometric mean N:P for leaves, stems and roots were 21.0 ± 1.3, 19.9 ± 1.4, and 24.3 ± 1.8, respectively, in legume shrubs, and 14.1 ± 1.4, 11.6 ± 1.6, and 9.8 ± 1.9, respectively, in non-N_2_-fixing shrubs. STN, STP and soil N:P (SNP) of the soil where legume shrubs rooted were 1.1 ± 2.2, 0.5 ± 1.7, and 2.3 ± 2.0, respectively, while those of soil where non-N_2_-fixing shrubs rooted were 1.4 ± 2.0, 0.5 ± 1.6, and 2.7 ± 1.9, respectively. Legume shrubs had higher [N] and N:P than non-N_2_-fixing shrubs for all tissues, but similar [P] (Figure 2). It was also noticeable that the ranges of [N], [P], and N:P were much larger in roots than in leaves for both functional types (Figure 2).

### Stoichiometric homeostasis of legume shrubs and non-N_2_-fixing shrubs

**Figure 3:**
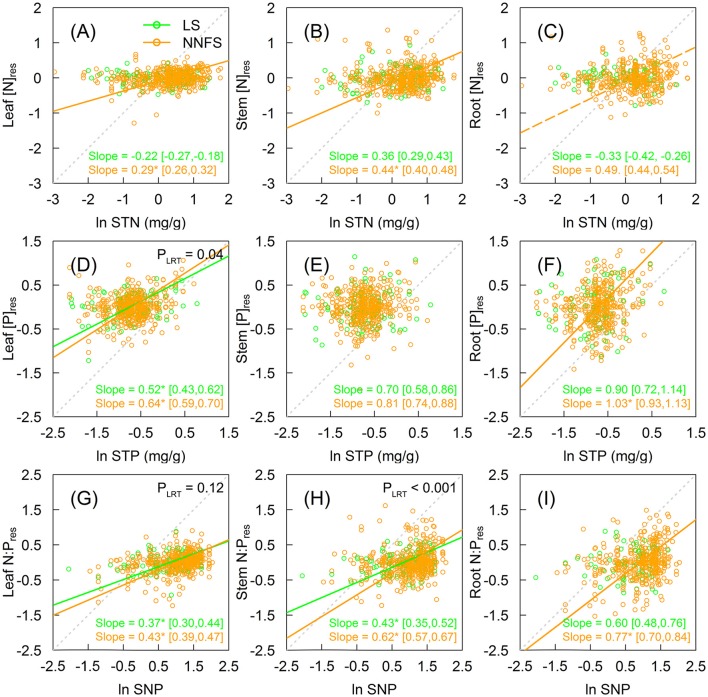
The relationship between soil and plant [N]_res_ **(A–C)**, [P]_res_ **(D–F)**, and N:P_res_ **(G–I)** in different tissues of legume shrubs (LS) (green) and non-N_2_-fixing shrubs (NNFS) (orange). The gray dotted lines represent the 1:1 lines. Solid lines and slopes followed by an asterisk show significant (*p* < 0.05), while the dashed lines and the slopes followed by a dot show marginally significant (0.05 < *p* < 0.1) relationships. The values in the brackets show 95% confidential intervals of the regression slopes. *P*-values of likelihood ratio tests (P_LRT_) are shown if both slopes are significant. STN, soil total nitrogen; STP, soil total phosphorus; SNP, soil N:P. [N]_res_, [P]_res_ and N:P_res_ are residuals of [N], [P] and N:P, respectively, after excluding effects of taxonomy and environmental variables.

[N]_res_ in legume shrubs did not show significant trends in all tissues as STN increased (Figures 3A–C). On the contrary, [N]_res_ in overall non-N_2_-fixing shrubs increased with STN, with slopes of 0.29 (95% CI: 0.26–0.32) (*H* = 3.45), 0.44 (95% CI: 0.40–0.48) (*H* = 2.27) and 0.49 (95% CI: 0.44–0.54) (*H* = 2.04) for leaves, stems, and roots, respectively, though the significance for roots was weaker (*p* = 0.07) (Figures 3A–C). The [N]_res_ also increased with STN for Rosaceae and *Vitex* for all tissues and the remaining non-N_2_-fixing shrubs for leaves (Supplementary Figures 1A–C). Unlike [N]_res_, legume shrubs and non-N_2_-fixing shrubs did not show clear patterns in relationships between [P]_res_ and STP (Figures 3D–F and Supplementary Figures 1D–F). For legume shrubs, N:P_res_ increased significantly with SNP in leaves (slope = 0.37, 95% CI: 0.30–0.44) (*H* = 2.70) and stems (slope = 0.43, 95% CI: 0.35–052) (*H* = 2.33), but not in roots (Figures 3G–I). For non-N_2_-fixing shrubs, N:P_res_ increased with SNP in all tissues (Figures 3G–I and Supplementary Figures 1G–I). Slopes between N:P_res_ and SNP of overall non-N_2_-fixing shrubs were 0.43 (95% CI: 0.39–0.47) (*H* = 2.33), 0.62 (0.57–0.67) (*H* = 1.61), and 0.77 (0.70–0.84) (*H* = 1.30) for leaves, stems, and roots, respectively (Figures 3G–I). Moreover, the slope in stems is significantly smaller in legume shrubs than in overall non-N_2_-fixing shrubs (0.43 vs. 0.62 in stems, with *p* < 0.05) (Figure 3H). *H* of [N]_res_ and N:P_res_ ratios of overall non-N_2_-fixing shrubs decreased from upper tissue type (leaves) to lower tissue type (roots) (Figures 3A–C, G–I).

### Nutrient scaling among tissue types of legume shrubs and non-N_2_-fixing shrubs

**Figure 4:**
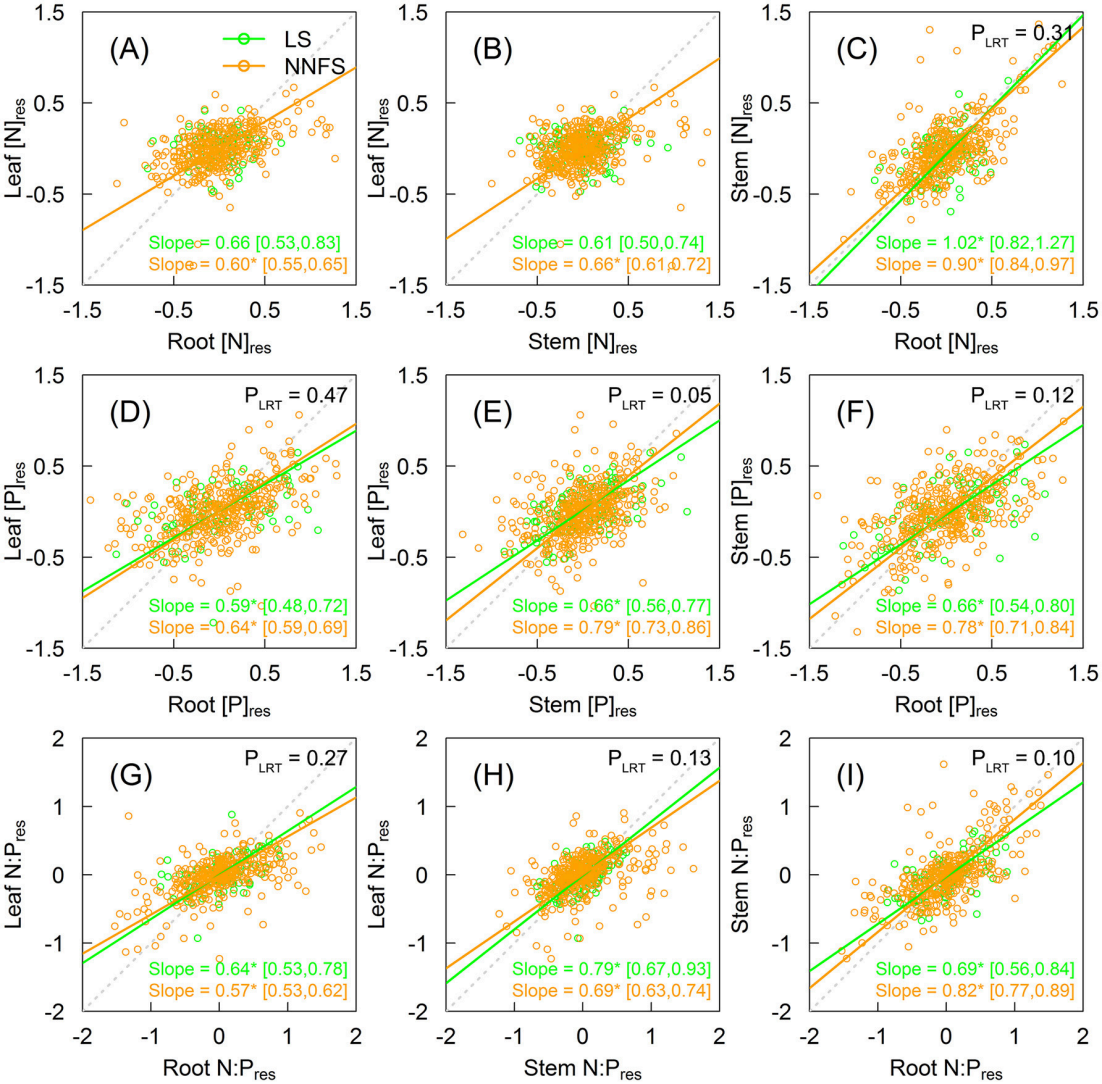
Scaling relationships of [N]_res_ **(A–C)**, [P]_res_ **(D–F)**, and N:P_res_ **(G–I)** among different tissues of legume shrubs (LS) (green) and non-N_2_-fixing shrubs (NNFS) (orange). The gray dotted lines represent the 1:1 lines. Solid lines and slopes followed by an asterisk show significant (*p* < 0.05) relationships. The values in the brackets show 95% confidential intervals of the regression slopes. *P*-values of likelihood ratio tests (P_*LRT*_) are shown if both slopes are significant. [N]_res_, [P]_res_ and N:P_res_ are residuals of [N], [P] and N:P, respectively, after excluding effects of taxonomy and environmental variables.

[N]_res_ in legume shrubs were not correlated between leaves and non-leaf tissues (stems or roots), but were correlated between stems or roots (slope = 1.02, 95% CI: 0.82–1.27) (Figures 4A–C). In contrast, [N]_res_ in non-N_2_-fixing shrubs were significantly correlated among leaves, stems and roots (Figures 4A–C and Supplementary Figures 2A–C), with exponential slopes of 0.60 (95% CI: 0.55–0.65), 0.66 (95% CI: 0.61–0.72), and 0.90 (95% CI: 0.84–0.97) for leaf-root, leaf-stem, and stem-root, respectively (Figures 4A–C). [P]_res_ and N:P_res_ were correlated across tissues for both legume shrubs and non-N_2_-fixing shrubs (Figures 4D–I and Supplementary Figures 2D–I). Legume shrubs and overall non-N_2_-fixing shrubs had similar slopes in [P]_res_ and N:P_res_ for all tissue pairs except between the leaf [P]_res_ and the stem [P]_res_ (0.66 for legume shrubs vs. 0.79 for overall non-N_2_-fixing shrubs, *p* = 0.046) (Figures 4D–I).

### N vs. P scaling within tissue types of legume shrubs and non-N_2_-fixing shrubs

**Figure 5:**
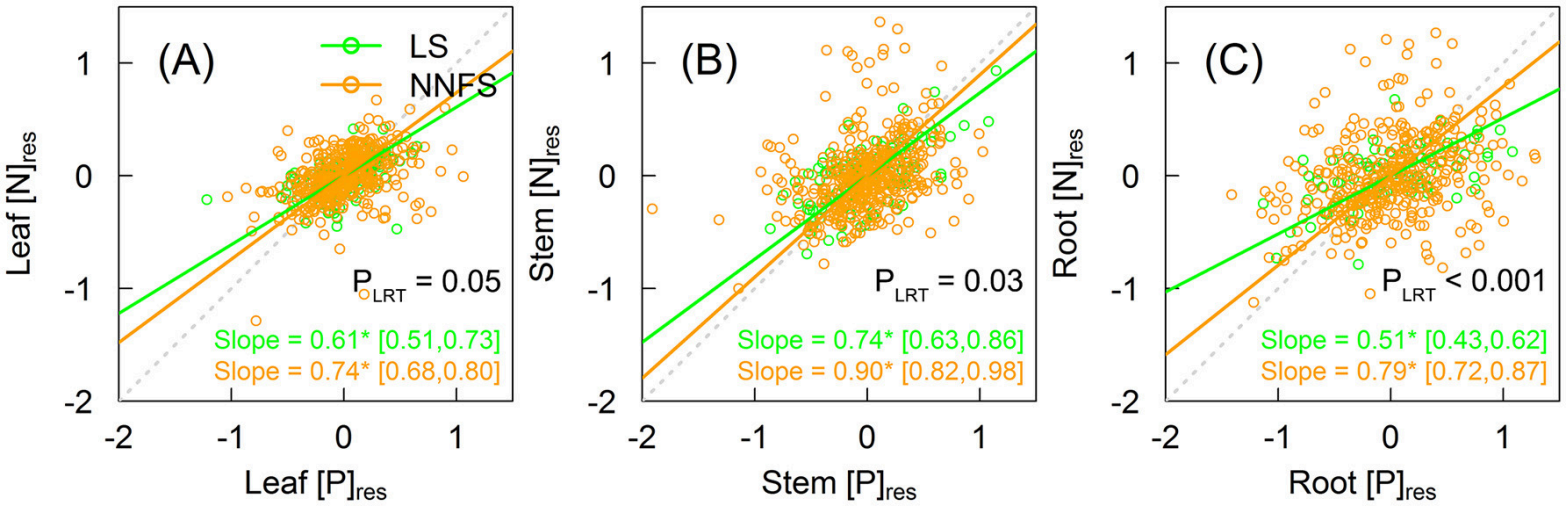
Scaling relationships between [N]_res_ and [P]_res_ within leaves **(A)**, stems **(B)**, and roots **(C)** of legume shrubs (LS) (green) and non-N_2_-fixing shrubs (NNFS) (orange). The gray dotted lines represent the 1:1 lines. Solid lines and slopes followed by an asterisk show significant (*p* < 0.05) relationships. The values in the brackets show 95% confidential intervals of the regression slopes. *P*-values of likelihood ratio tests (P_LRT_) are shown if both slopes are significant. [N]_res_ and [P]_res_, residuals of [N] and [P], respectively, after excluding effects of taxonomy and environmental variables.

[N]_res_ and [P]_res_ were positively correlated within three tissue types for both legume shrubs and overall non-N_2_-fixing shrubs (Figure 5). The slopes were 0.61 (95% CI: 0.51–0.73), 0.74 (95% CI: 0.63–0.86), and 0.51 (95% CI: 0.43–0.62) for leaves, stems and roots in legume shrubs, and 0.74 (95% CI: 0.68–0.80), 0.90 (95% CI: 0.82–0.98), and 0.79 (95% CI: 0.72–0.87) for leaves, stems and roots in overall non-N_2_-fixing shrubs, respectively (Figure 5). Slopes were flatter in legume shrubs than in overall non-N_2_-fixing shrubs (0.61 vs. 0.74 for leaves, 0.74 vs. 0.90 for stems, and 0.51 vs. 0.79 for roots, all with *p* < 0.05) (Figure 5). Compared to legume shrubs, slopes were steeper in stems and roots for the Rosaceae, in leaves for the *Vitex*, and in all tissue types for the remaining non-N_2_-fixing shrubs (Supplementary Figure 3).

## Discussion

In this study, we compared N stoichiometry of legume shrubs and non-N_2_-fixing shrubs to explore the potential influence of SNF on plant stoichiometry. The results showed that N_2_-fixing legume shrubs were richer in N, more homeostatic and more independent in N correlations among tissues, but had larger demand for P, than non-N_2_-fixing shrubs. The patterns also hold when comparing legume shrubs to a single family (Rosaceae), or a single genus (*Vitex*) of non-N_2_-fixing shrubs or to the remaining non-N_2_-fixing shrubs, strongly supporting the hypothesis that SNF provided higher [N] and stronger homeostasis for N in legume shrubs compared to non-N_2_-fixing shrubs in general. Consequently, higher [N] and stronger homeostasis resulted in weaker N co-variation among tissues of legume shrubs. Nevertheless, owing to the extraordinarily high N:P and flat N–P slopes, legume shrubs need more P to balance their N income. Detailed comparisons between these two functional groups and influences of SNF on stoichiometry of legume shrubs are discussed below.

### Nutrient concentrations and ratios in legume shrubs and non-N_2_-fixing shrubs

The comparison results support our first hypothesis about the nutrient concentrations and ratios. [N] and N:P in three tissues were higher in legume shrubs than in non-N_2_-fixing shrubs, but STN and SNP were even lower in microsites with legume shrubs than in microsites with non-N_2_-fixing shrubs (Table 1), suggesting that SNF provides legume shrubs with a clear N advantage compared to non-N_2_-fixing shrubs. Studies in tropical forests suggested that legume plants also have P advantage in addition to N, owing to their production of N-rich phosphatase to break down organic P (Houlton et al., 2008; Nasto et al., 2014). However, we did not find higher [P] in legume shrubs probably because the strategy of temperate shrubs is different. Another possible reason is that the relatively more P-limited soil in China (Han et al., 2005) suppressed the P advantage of legume shrubs, and the high leaf N:P (Table 1) also suggests that legume shrubs could face strong P limitation (Güsewell, 2004).

**Table 1:** Comparison of nutrient composition between legume shrubs and non-N_2_-fixing shrubs.

**Variable**	**No. (legume shrubs vs. non-N_2_-fixing shrubs)**	**Geometric mean ± geometric *SD* (legume shrubs vs. non-N_2_-fixing shrubs)**	***P*-value (*F*-test)**	***P*-value (*t*-test)**
Leaf [N]	105 vs. 530	26.7 ± 1.2 vs. 18.3 ± 1.3 mg.g^−1^	0.01	0.00
Stem [N]	102 vs. 522	9.43 ± 1.5 vs. 5.28 ± 1.5 mg.g^−1^	0.46	0.00
Root [N]	76 vs. 452	12.6 ± 1.5 vs. 5.73 ± 1.6 mg.g^−1^	0.10	0.00
STN	105 vs. 528	1.08 ± 2.2 vs. 1.36 ± 2.0 mg.g^−1^	0.11	0.00
Leaf [P]	105 vs. 530	1.27 ± 1.4 vs. 1.29 ± 1.4 mg.g^−1^	0.32	0.58
Stem [P]	102 vs. 522	0.47 ± 1.5 vs. 0.46 ± 1.5 mg.g^−1^	0.30	0.41
Root [P]	76 vs. 452	0.52 ± 1.7 vs. 0.58 ± 1.7 mg.g^−1^	0.73	0.08
STP	105 vs. 528	0.47 ± 1.7 vs. 0.50 ± 1.6 mg.g^−1^	0.01	0.25
Leaf N:P	105 vs. 530	21.0 ± 1.3 vs. 14.1 ± 1.4	0.00	0.00
Stem N:P	102 vs. 522	19.9 ± 1.4 vs. 11.6 ± 1.6	0.00	0.00
Root N:P	76 vs. 452	24.3 ± 1.8 vs. 9.82 ± 1.9	0.16	0.00
SNP	105 vs. 528	2.28 ± 2.0 vs. 2.70 ± 1.9	0.34	0.01

*SD, standard deviation; STN, soil total nitrogen; STP, soil total phosphorus; SNP, soil N:P*.

### Stoichiometric homeostasis of legume shrubs and non-N_2_-fixing shrubs

Strict stoichiometric homeostasis in N was evident for legume shrubs, but not for non-N_2_-fixing shrubs. In agreement with our second hypothesis, STN does not affect [N], indicating strict homeostasis in N, in different tissue types of legume shrubs. SNF provides an additional N supply, which offsets N limitation and allows legume shrubs to keep stable [N] (Lambers et al., 2008; Hobbie, 2015). Therefore, legume shrubs are able to maintain physiological function and gain higher fitness than other plants under N-limited conditions, so that they can have higher dominance on barren soil (Rastetter et al., 2001; Menge et al., 2008). Due to the influence of N homeostasis, N:P_res_ were also more homeostatic in legume shrubs than in non-N_2_-fixing shrubs (Figures 3G–I). In addition, N:P_res_ were only correlated with [P]_res_ but not [N]_res_ in legume shrubs, whereas N:P_res_ were both correlated with [N]_res_ and [P]_res_ in non-N_2_-fixing shrubs (Supplementary Figure 4), suggesting the contribution of N homeostasis to the stability of N in legume shrubs.

Consistent with previous studies (Yu et al., 2010; Sardans et al., 2016), soil N availability improved [N] of non-N_2_-fixing shrubs. However, such effects might be masked by the effects of taxonomy and other environmental factors (Yang et al., 2016). The stronger stoichiometric homeostasis in leaves corresponds to previous studies with other plants (Garrish et al., 2010; Schreeg et al., 2014; Yan et al., 2016). Leaf [N] is closely related to many important physiological activities, such as photosynthesis and respiration (Reich et al., 2003), so plants maintain high and stable [N] in leaves to keep the efficiency of necessary metabolic processes (Güsewell, 2004). Similarly, the more constrained N:P in leaves also reflects that leaves are less responsive to the variation of soil nutrient composition because of the demand to keep efficient carbon fixation (Sterner and Elser, 2002; Schreeg et al., 2014).

In a previous study, Brouwer (1983) proposed that nutrients were transported to distant tissues only after the tissues closer to the nutrient source had met their needs. Based on this hypothesis, the closer the tissue is to the nutrient source, the higher its priority to nutrients will be. In other words, we would expect that the variation of soil nutrients would affect roots most and leaves least. Indeed, we found a steeper slope between STN and root [N]_res_ than between STN and other tissue types' [N]_res_, and the slope of the root N:P_res_-SNP relationship was also steeper than between SNP and other tissue types' N:P_res_ in non-N_2_-fixing shrubs. Our results suggest that changes in root nutrient composition can mirror soil's nutrient variation better than other tissues.

[P]_res_ did not show clear patterns with STP for either legume shrubs or non-N_2_-fixing shrubs, probably because soil available P is highly sensitive to the environment (He and Dijkstra, 2014) and STP may not be a good surrogate for soil P availability.

### Nutrient scaling among tissue types in legume shrubs and non-N_2_-fixing shrubs

The correlations of nutrients among tissue types were stronger in non-N_2_-fixing shrubs than in legume shrubs (Figure 4), after controlling for other factors. Again, the difference may be masked by the effects of taxonomy and environments (Yang et al., 2014). STN was the only driving force of N variation among tissues in our case. [N]_res_ in each tissue in non-N_2_-fixing shrubs responded to STN in a similar fashion (Figures 3A–C), so we could observe positive [N]_res_ relationships. In contrast, [N]_res_ in legume shrubs were homeostatic to STN (Figures 3A–C), owing to sufficient N supply independent of the soil N pool, and thus they showed weaker [N]_res_ co-variation among tissue types. However, the positive [N]_res_ relationship still existed between structural tissue types (stem and root), probably due to their physiological similarity (Kerkhoff et al., 2006).

Unlike [N]_res_, we found the coordinated [P]_res_ and N:P_res_ among tissues in legume shrubs (Figures 4D–I), still demonstrating physiological and ecological connections among tissues (Kerkhoff et al., 2006) for legume shrubs and non-N_2_-fixing shrubs. The synchronized variation of nutrients among leaves, stems and roots is consistent with previous studies across various plant species (Kerkhoff et al., 2006; Yang et al., 2014). The scaling slopes of [N]_res_ (non-N_2_-fixing shrubs) and [P]_res_ (both legume shrubs and non-N_2_-fixing shrubs) showed that tissues further away from the soil accumulated nutrients more slowly than those closer to the soil, supporting Brouwer's hypothesis that tissue types nearest to the nutritional source are preferential in nutrient allocation (Brouwer, 1983; Yang et al., 2014). Furthermore, tissues further away from the soil had a reduced nutrient variation rate, implying stronger nutrient homeostasis, than those closer to the soil (Yan et al., 2016), in agreement with our results for homeostasis. The scaling slopes of N:P_res_ among tissue types in legume shrubs and non-N_2_-fixing shrubs also followed the same pattern mentioned above, suggesting that leaves are more stable in nutrient ratio than stems and roots (Schreeg et al., 2014; Yan et al., 2016).

### N vs. P scaling within tissue types of legume shrubs and non-N_2_-fixing shrubs

Consistent with our fourth hypothesis, we found lower N–P slopes in each tissue type in legume shrubs. Correlated N and P scaling relationships of non-N_2_-fixing shrubs can be partly explained by a strong correlation between ln-transformed STN and STP (R^2^ = 0.20) (Güsewell and Koerselman, 2002; He et al., 2008). Our results concur with prior studies that P accumulates faster than N in leaves (Wright et al., 2004; Elser et al., 2010), as well as in stems and roots (Kerkhoff et al., 2006). However, the slopes in this study are not comparable with other studies because residuals (rather than real concentration data) were used in this study. P limitation can reduce the slope, but N limitation will do the opposite (Ågren, 2008). The slopes were flatter in legume shrubs than in non-N_2_-fixing shrubs (Figure 5), suggesting that P increases faster in legume shrubs than non-N_2_-fixing shrubs and that legume shrubs are more P-limited due to excess N absorption of SNF (Ågren, 2008). Meanwhile, higher plant N:P also demonstrates that P limitation is stronger in legume shrubs than non-N_2_-fixing shrubs (Güsewell, 2004). Besides requiring more P to maintain optimal N:P, legume shrubs also need enough P to maintain the functioning of the SNF (Vitousek and Field, 1999; Benner and Vitousek, 2007; Sulieman and Tran, 2015). Therefore, N_2_-fixing legume shrubs demand more P and may be consequently more P-limited than non-N_2_-fixing shrubs (Vitousek et al., 2010). The differences between N–P slopes (Figure 5) and between N:P ratios (Table 1) were smaller in leaves than in other tissue types, suggesting the stronger stoichiometric stability in leaves to sustain normal physiology (Schreeg et al., 2014; Yan et al., 2016).

In conclusion, there are obvious stoichiometric differences between legume shrubs and non-N_2_-fixing shrubs. Extra N supply in legume shrubs enhances their homeostasis to soil nutrient deficiency and provides stronger N stability. However, due to the surplus uptake of N, legume shrubs may suffer from potential P limitation. Legume shrubs are good nurse plants at the early succession stage of restoration habitats, which can provide not only canopy shade but also more N (Gómez-Aparicio et al., 2004; Zhao et al., 2007). Nevertheless, special attention should be paid to the nutrient conditions of nurse legumes, due to their tendency of being P-limited. We were not able to measure the actual SNF, so further studies on how these differences are connected with SNF and plant fitness are now necessary to unravel the underlying evolutionary and physiological mechanisms.

## Author contributions

ZT and YG designed the research; ZT and XY collected data; YG analyzed data. YG, XY, CS, YJ, and ZT wrote the manuscript and gave final approval for publication.

### Conflict of interest statement

The authors declare that the research was conducted in the absence of any commercial or financial relationships that could be construed as a potential conflict of interest.
