# Supplementary material for: Legume Shrubs Are More Nitrogen-Homeostatic than Non-legume Shrubs
Source: Front Plant Sci. 2017 Sep 26;8:1662. doi: 10.3389/fpls.2017.01662 (PMC5622988; doi:10.3389/fpls.2017.01662)
Supplement: Supplementary file 1 [file DataSheet1.docx]

***Supplementary Material***

**Legume shrubs are more nitrogen-homeostatic than non-legume shrubs**

**Yanpei Guo^1,2^, Xian Yang^1,3^, Christian** **Schöb^2,5^, Youxu Jiang^1,4^, Zhiyao Tang^1*^**

^1^ Department of Ecology, College of Urban and Environmental Sciences and Key Laboratory for Earth Surface Processes, Peking University, Beijing, China

^2^ Department of Evolutionary Biology and Environmental Studies, University of Zurich, Zurich, Switzerland

^3^ School of Biology, Georgia Institute of Technology, Atlanta, GA, USA

^4^ Institute of Forest Ecological Environment and Protection, Chinese Academy of Forestry, Beijing, China

^5^ Present address: Department of Environmental Systems Science, Swiss Federal Institute of Technology, ETH Zurich, Zurich, Switzerland

^*^ **Correspondence**:

Dr. Zhiyao Tang

zytang@urban.pku.edu.cn

**Supplementary Figures**


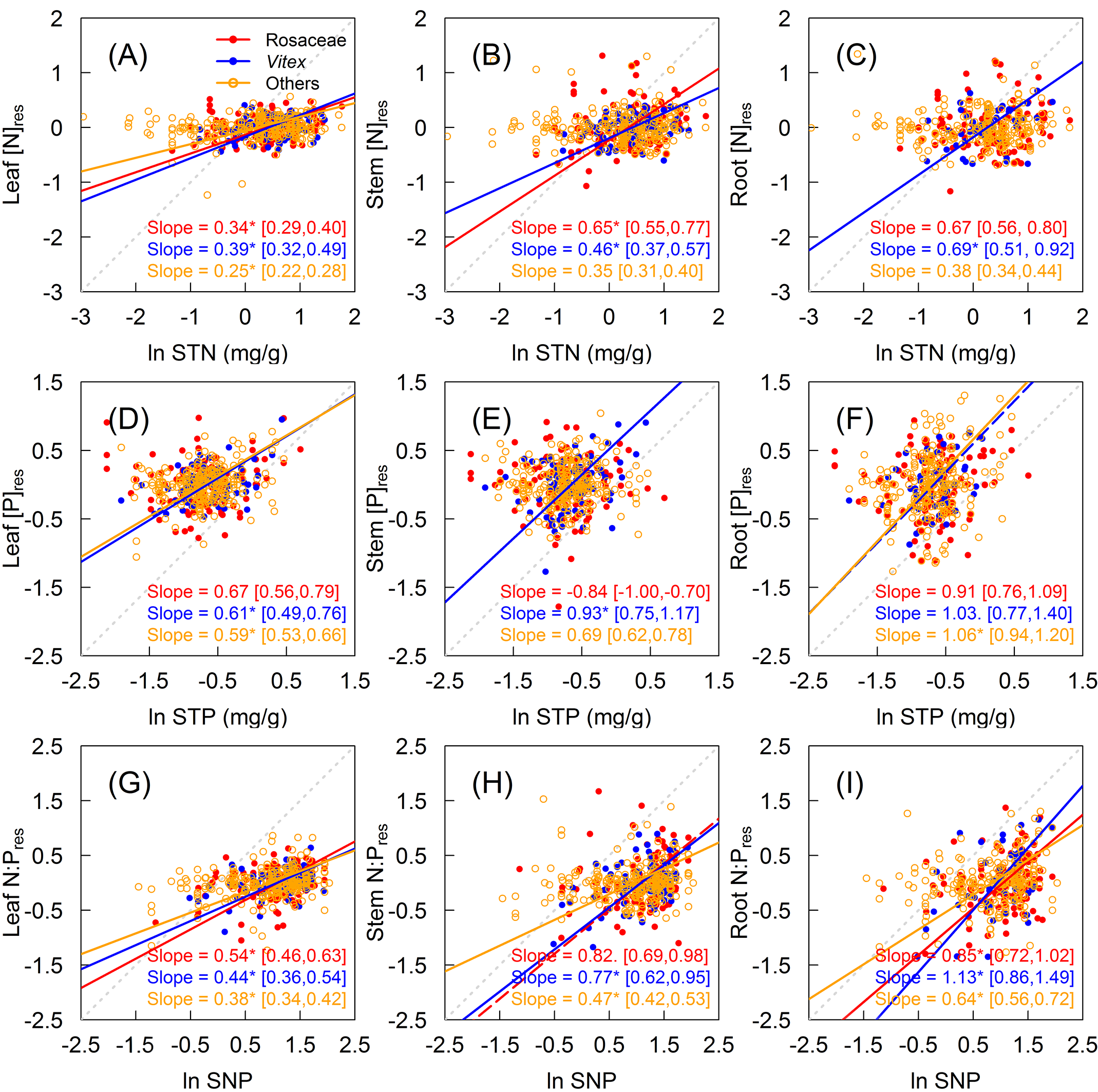


**Supplementary Figure 1.** The relationship between soil and plant [N]_res_ (A-C), [P]_res_ (E-F) and N:P_res_ (G-I) in different tissues of species from the Rosaceae (red), the *Vitex* (blue), and the remaining non-N-fixers (orange). The grey dotted lines represent the 1:1 lines. Solid lines and slopes followed by an asterisk show significant (p < 0.05), while the dashed lines and the slopes followed by a dot show marginally significant (0.05 < p < 0.1) relationships. The values in the brackets show 95% confidential intervals of the regression slopes. STN, soil total nitrogen; STP, soil total phosphorus; SNP, soil N:P. [N]_res_, [P]_res_ and N:P_res_, residuals of [N], [P] and N:P, respectively, after excluding effects of taxonomy and environmental variables.


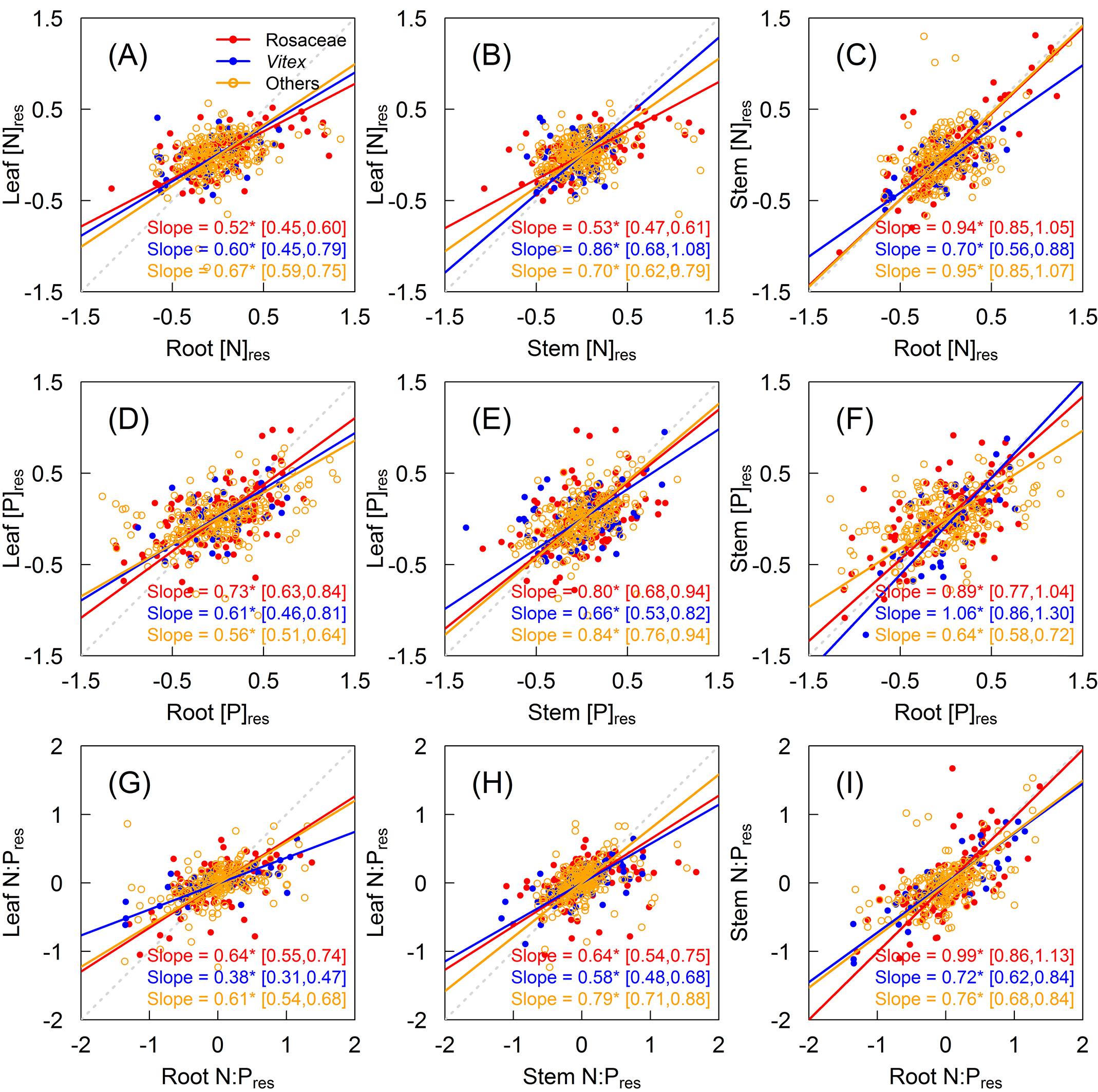


**Supplementary Figure 2.** Scaling relationships of [N]_res_ (A-C), [P]_res_ (D-F) and N:P_res_ (G-I) among different tissues of species from the Rosaceae (red), the *Vitex* (blue), and the remaining non-N-fixers (orange). The 1:1 lines are shown as grey dotted lines. Solid lines and slopes followed by an asterisk show significant (p < 0.05) relationships. The values in the brackets show 95% confidential intervals of the regression slopes. [N]_res_, [P]_res_ and N:P_res_, residuals of [N], [P] and N:P, respectively, after excluding effects of taxonomy and environmental variables.


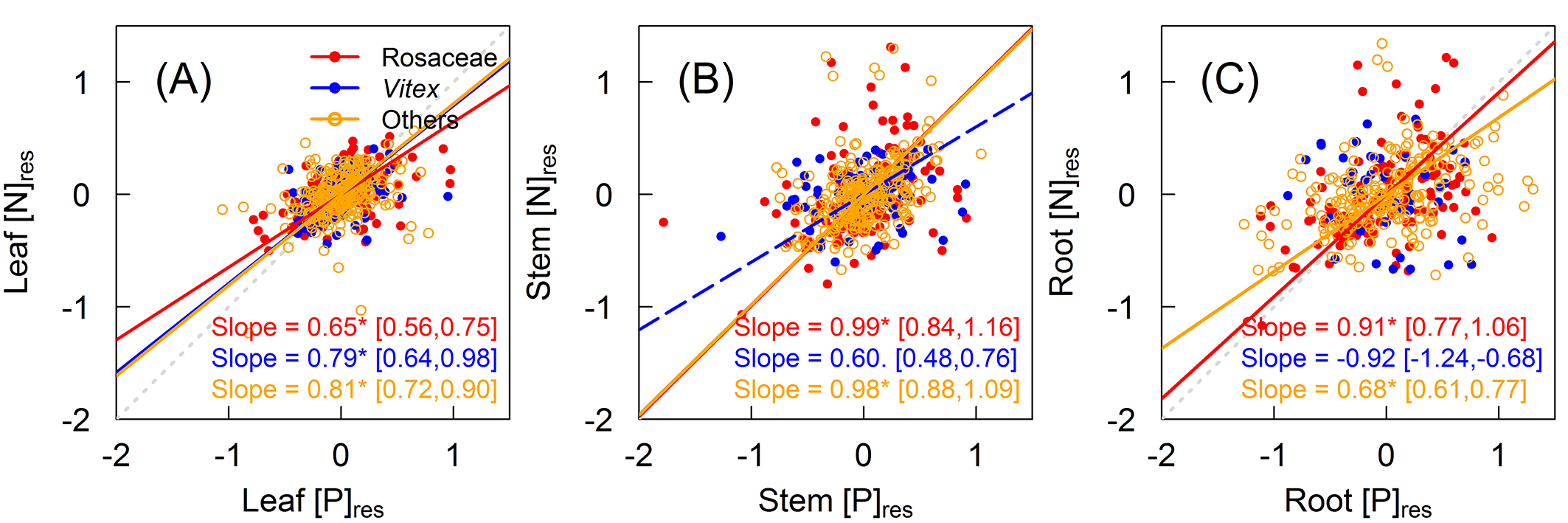


**Supplementary Figure 3.** Scaling relationships between [N]_res_ and [P]_res_ within leaves (A), stems (B) and roots (C) of species from the Rosaceae (red), the *Vitex* (blue), and the remaining non-N-fixers (orange). The 1:1 lines are shown as grey dotted lines. Solid lines and slopes followed by an asterisk show significant (p < 0.05), while the dashed lines and the slopes followed by a dot show marginally significant (0.05 < p < 0.1) relationships. The values in the brackets show 95% confidential intervals of the regression slopes. [N]_res_ and [P]_res_, residuals of [N] and [P], respectively, after excluding effects of taxonomy and environmental variables.


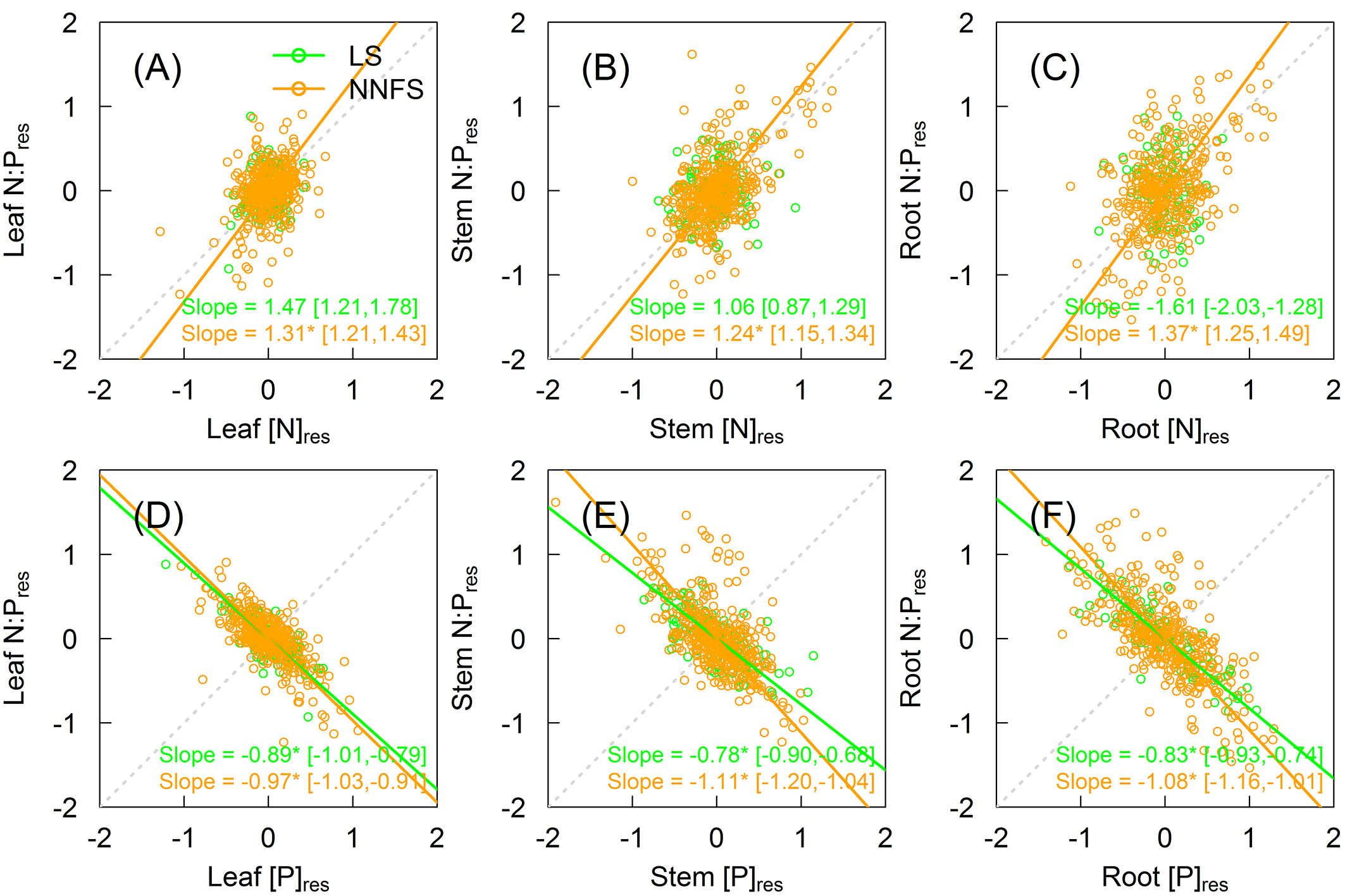


**Supplementary Figure 4.** The relationship between N:P and plant N (A-C) and P (D-F) in different tissues of legumes (green) and non-N-fixers (orange). The 1:1 lines are shown as grey dotted lines. Solid lines and slopes followed by an asterisk show significant (p < 0.05) relationships. 95% confidential intervals of regression slopes are also shown in the brackets.
